# (1-Acetyl­thio­urea-κ*S*)bromido­bis(triphenyl­phosphane-κ*P*)silver(I)

**DOI:** 10.1107/S1600536812045199

**Published:** 2012-11-17

**Authors:** Chaveng Pakawatchai, Piyapong Jantaramas, Jedsada Mokhagul, Ruthairat Nimthong

**Affiliations:** a Department of Chemistry, Faculty of Science, Prince of Songkla University, Hat Yai, Songkhla 90112, Thailand; b Department of Chemistry and Center for Innovation in Chemistry, Faculty of Science, Prince of Songkla University, Hat Yai, Songkhla 90112, Thailand

## Abstract

In the title complex, [AgBr(C_3_H_6_N_2_OS)(C_18_H_15_P)_2_], the Ag^I^ ion is in a distorted tetra­hedral geometry coordinated by two P atoms from two triphenyl­phosphane ligands, one S atom of an acetyl­thio­urea ligand and one bromide ligand. There are intra­molecular N—H⋯Br and N—H⋯O hydrogen bonds present. In the crystal, pairs of N—H⋯S hydrogen bonds involving thio­urea groups form inversion dimers. In addition, moleclues pack to give sixfold phenyl embraces with an inter­molecular P⋯P distance of 6.4586 (17) Å.

## Related literature

For the definition of sixfold phenyl embraces, see: Dance & Scudder(2000 ▶). For the synthesis and structure of silver(I) coordination compounds and their potential applications, see: Ferrari *et al.* (2007 ▶); Lobana *et al.* (2008 ▶); Isab *et al.* (2010 ▶); Nawaz *et al.* (2011 ▶). For relevant examples of discrete complexes, see: Aslanidis *et al.* (1997 ▶); Nomiya *et al.* (1998 ▶); Lobana *et al.* (2008 ▶); Zhang *et al.* (2008 ▶).
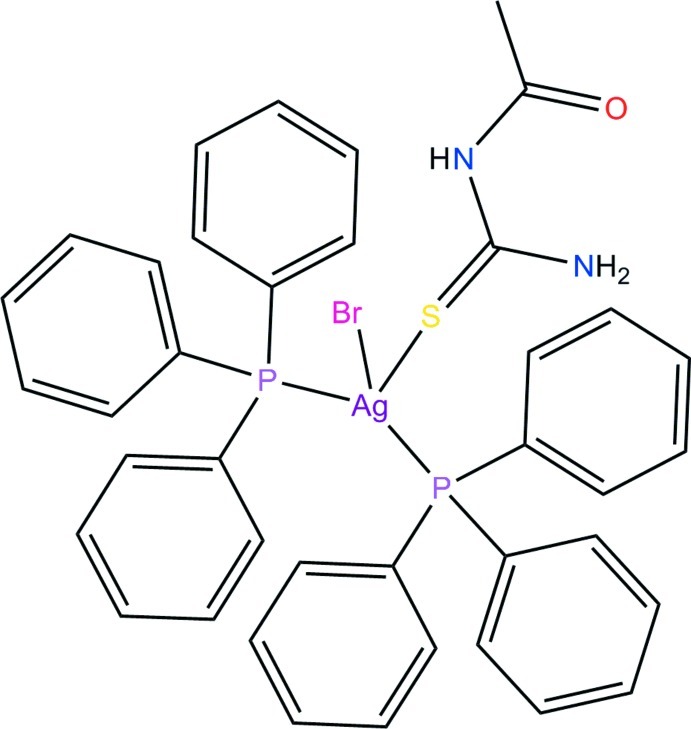


## Experimental

### 

#### Crystal data

[AgBr(C_3_H_6_N_2_OS)(C_18_H_15_P)_2_]
*M* *_r_* = 830.48
Triclinic, 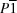

*a* = 10.4684 (12) Å
*b* = 12.9898 (14) Å
*c* = 14.8354 (16) Å
α = 71.091 (2)°
β = 80.955 (3)°
γ = 72.261 (2)°
*V* = 1813.9 (3) Å^3^
*Z* = 2
Mo *K*α radiation
μ = 1.84 mm^−1^
*T* = 293 K
0.23 × 0.11 × 0.02 mm

#### Data collection

Bruker SMART CCD diffractometer
Absorption correction: multi-scan (*SADABS*; Bruker, 2003 ▶) *T*_min_ = 0.793, *T*_max_ = 0.957
25247 measured reflections
8788 independent reflections
6789 reflections with *I* > 2σ(*I*)
*R*_int_ = 0.046

#### Refinement

*R*[*F* ^2^ > 2σ(*F* ^2^)] = 0.048
*wR*(*F* ^2^) = 0.099
*S* = 1.07
8788 reflections
434 parameters
H atoms treated by a mixture of independent and constrained refinement
Δρ_max_ = 0.86 e Å^−3^
Δρ_min_ = −0.47 e Å^−3^

### 

Data collection: *SMART* (Bruker, 1998 ▶); cell refinement: *SAINT* (Bruker, 2003 ▶); data reduction: *SAINT*; program(s) used to solve structure: *SHELXS97* (Sheldrick, 2008 ▶); program(s) used to refine structure: *SHELXL97* (Sheldrick, 2008 ▶); molecular graphics: *Mercury* (Macrae, 2008) ▶; software used to prepare material for publication: *SHELXL97* and *publCIF* (Westrip, 2010 ▶).

## Supplementary Material

Crystal structure: contains datablock(s) I, global. DOI: 10.1107/S1600536812045199/lh5550sup1.cif

Structure factors: contains datablock(s) I. DOI: 10.1107/S1600536812045199/lh5550Isup2.hkl

Additional supplementary materials:  crystallographic information; 3D view; checkCIF report

## Figures and Tables

**Table 1:** Hydrogen-bond geometry (Å, °)

*D*—H⋯*A*	*D*—H	H⋯*A*	*D*⋯*A*	*D*—H⋯*A*
N1—H1*A*⋯S^i^	0.84 (4)	2.74 (4)	3.524 (4)	158 (3)
N1—H1*B*⋯O	0.84 (4)	1.99 (4)	2.642 (5)	135 (4)
N2—H2⋯Br	0.89 (4)	2.52 (4)	3.402 (3)	174 (3)

Symmetry code: (i) 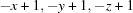.

**Fig. 1.**
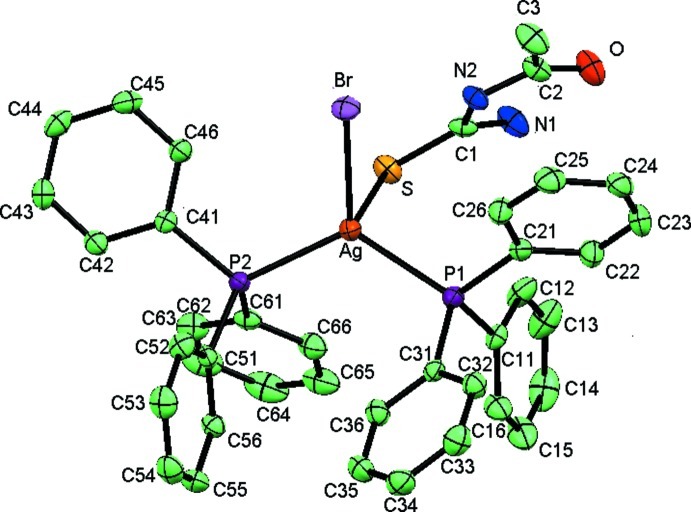
The molecular structure with displacement ellipsoids drawn at the 50% probability level.

**Fig. 2.**
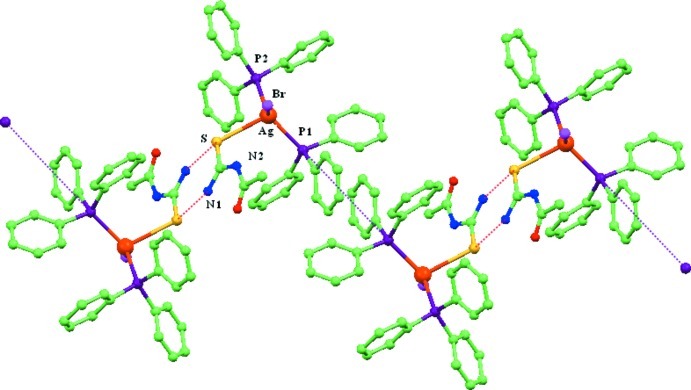
Part of the crystal structure showing intermolecular hydrogen (red dashed lines) bond and six fold phenyl embraces shown as purple dashed lines.

## supplementary crystallographic information

### Comment

The studies of silver(I) complexes with tertiary phosphane and sulfur donor ligands as co-ligainds has progressed extensively in recent years (Lobana *et al.*, 2008; Nawaz *et al.*, 2011) because of their potential applications such as antimicrobial activities (Isab *et al.*, 2010) and they also often show interesting luminescence properties (Ferrari *et al.*, 2007). Moreover, sixfold phenyl embraces (6PE), a common motif of the six phenyl groups of two adjacent triphenylphosphane (PPh_3_) ligands have been also widely studied, where six phenyl rings in the interaction zone participate in a concerted cycle of edge-to-face (ef) phenyl···phenyl interactions (Dance *et al.*, 2000).

The molecular structure of the title compound (I) is shown in Fig. 1. In the mononuclear complex, the Ag^I^ ion exists in a distorted tetrahedral geometry. The Ag—P1 and Ag—P2 distances of 2.4807 (9) and 2.4657 (9) Å are closed to the values of [AgBr(–S-Hpytsc(Ph_3_P)_2_].CH_3_CN (Ag—P1 = 2.4605 (19), Ag—P2 = 2.4926 (19) Å) (Lobana *et al.*, 2008). The observed Ag—S distance of 2.8789 (10) Å in (I) is appreciably longer than the mean value of 2.632 (1) Å for [Ag(PPh_3_)_2_(pytH)_2_]NO_3_ (Aslanidis *et al.*, 1997). The P1—Ag—P2 angle of 124.52 (3)° approaches close to the average value found in compounds containing an Ag^I^ ion bound to two triphenylphosphanes *e.g.* in [Ag(1,2,4-*L*)(PPh_3_)_2_]_n_ (HL = triazole) (P2—Ag—P1 = 126.29 (7)°) (Nomiya *et al.*, 1998) and in [(Ph_3_P)_2_AgO_3_SCH_3_] (P2—Ag—P1 = 132.4 (4)°) (Zhang *et al*.,2008). There are intramolecular N2—H···Br and N1—H···O hydrogen bonds present. In the crystal, N and S atoms of the thiourea groups are invovled in forming hydrogen bonded dimers across an inversion center (symmetry code: -*x* + 1, -*y* + 1, -*z* + 1) and sixfold phenyl embraces with an intermolecular P···P distance of 6.4586 (17) Å are arranged in one-dimensional chains (Fig. 2).

### Experimental

Triphenylphosphane (0.28 g, 1.00 mmol) was dissolved in 30 cm^3^ of mixed sovents of acetonitrile and methanol at 343–348 K and then AgBr (0.10 g, 0.50 mmol) was added. The mixture was stirred for 2 h during that time a greenish precipitate was formed. Acetylthiourea (0.13 g, 1.00 mmol) was added and the new reaction mixture was heated under reflux for 5 h where upon the precipitate gradually disappeared. The resulting clear solution was filtered off and left to evaporate at room temperature. The crystalline solids, which were deposited upon standing for several days, were filtered off and dried in vacuo. Analysis found: C 57.58, H 4.09, N 3.27, S 2.37%; calculated for C39H32AgBrN2OP2S: C 56.40, H 4.37, N 3.37, S 3.86%.

### Refinement

The H atoms bonded to C atoms were constrained with a riding model of 0.93 Å (aryl H), and *U*_iso_(H) = 1.2*U*_eq_(C); 0.96 Å(CH_3_) and *U*_iso_(H) = 1.5*U*_eq_(C). All H atom bonded to the N atom was located in a difference Fourier map and refined isotropically.

### Crystal data

**Table:**

[AgBr(C_3_H_6_N_2_OS)(C_18_H_15_P)_2_]	*Z* = 2
*M**_r_* = 830.48	*F*(000) = 840
Triclinic, *P*1	*D*_x_ = 1.521 Mg m^−^^3^
Hall symbol: -P 1	Mo *K*α radiation, λ = 0.71073 Å
*a* = 10.4684 (12) Å	Cell parameters from 4019 reflections
*b* = 12.9898 (14) Å	θ = 2.3–21.9°
*c* = 14.8354 (16) Å	µ = 1.84 mm^−^^1^
α = 71.091 (2)°	*T* = 293 K
β = 80.955 (3)°	Hexagon, colorless
γ = 72.261 (2)°	0.23 × 0.11 × 0.02 mm
*V* = 1813.9 (3) Å^3^	

### Data collection

**Table:**

Bruker SMART CCD diffractometer	8788 independent reflections
Radiation source: fine-focus sealed tube	6789 reflections with *I* > 2σ(*I*)
Graphite monochromator	*R*_int_ = 0.046
Frames, each covering 0.3 ° in ω scans	θ_max_ = 28.1°, θ_min_ = 1.5°
Absorption correction: multi-scan (*SADABS*; Bruker, 2003)	*h* = −13→13
*T*_min_ = 0.793, *T*_max_ = 0.957	*k* = −17→17
25247 measured reflections	*l* = −19→19

### Refinement

**Table:**

Refinement on *F*^2^	Primary atom site location: structure-invariant direct methods
Least-squares matrix: full	Secondary atom site location: difference Fourier map
*R*[*F*^2^ > 2σ(*F*^2^)] = 0.048	Hydrogen site location: inferred from neighbouring sites
*wR*(*F*^2^) = 0.099	H atoms treated by a mixture of independent and constrained refinement
*S* = 1.07	*w* = 1/[σ^2^(*F*_o_^2^) + (0.0377*P*)^2^ + 0.3327*P*] where *P* = (*F*_o_^2^ + 2*F*_c_^2^)/3
8788 reflections	(Δ/σ)_max_ = 0.006
434 parameters	Δρ_max_ = 0.86 e Å^−^^3^
0 restraints	Δρ_min_ = −0.47 e Å^−^^3^

### Special details

**Table:**

Geometry. All e.s.d.'s (except the e.s.d. in the dihedral angle between two l.s. planes) are estimated using the full covariance matrix. The cell e.s.d.'s are taken into account individually in the estimation of e.s.d.'s in distances, angles and torsion angles; correlations between e.s.d.'s in cell parameters are only used when they are defined by crystal symmetry. An approximate (isotropic) treatment of cell e.s.d.'s is used for estimating e.s.d.'s involving l.s. planes.
Refinement. Refinement of *F*^2^ against ALL reflections. The weighted *R*-factor *wR* and goodness of fit *S* are based on *F*^2^, conventional *R*-factors *R* are based on *F*, with *F* set to zero for negative *F*^2^. The threshold expression of *F*^2^ > σ(*F*^2^) is used only for calculating *R*-factors(gt) *etc*. and is not relevant to the choice of reflections for refinement. *R*-factors based on *F*^2^ are statistically about twice as large as those based on *F*, and *R*- factors based on ALL data will be even larger.

### Fractional atomic coordinates and isotropic or equivalent isotropic displacement parameters (Å^2^)

**Table:**

	*x*	*y*	*z*	*U*_iso_*/*U*_eq_	
C1	0.2921 (3)	0.4815 (3)	0.4632 (2)	0.0363 (8)	
C2	0.1877 (4)	0.3499 (3)	0.4337 (3)	0.0442 (9)	
C3	0.0634 (4)	0.3492 (4)	0.3968 (3)	0.0606 (12)	
H3A	0.0345	0.4173	0.3458	0.091*	
H3B	−0.0062	0.3450	0.4473	0.091*	
H3C	0.0819	0.2849	0.3735	0.091*	
C11	0.4784 (3)	0.5721 (3)	0.1742 (2)	0.0351 (7)	
C12	0.5091 (4)	0.4991 (4)	0.2650 (3)	0.0578 (11)	
H12	0.4436	0.4688	0.3053	0.069*	
C13	0.6367 (5)	0.4717 (4)	0.2953 (3)	0.0714 (14)	
H13	0.6566	0.4238	0.3563	0.086*	
C14	0.7332 (5)	0.5144 (4)	0.2361 (4)	0.0726 (15)	
H14	0.8183	0.4977	0.2572	0.087*	
C15	0.7052 (4)	0.5817 (4)	0.1460 (4)	0.0688 (14)	
H15	0.7726	0.6080	0.1049	0.083*	
C16	0.5786 (4)	0.6112 (3)	0.1150 (3)	0.0498 (10)	
H16	0.5608	0.6580	0.0534	0.060*	
C21	0.2525 (3)	0.5013 (3)	0.1451 (2)	0.0354 (8)	
C22	0.3421 (4)	0.3957 (3)	0.1521 (3)	0.0504 (10)	
H22	0.4314	0.3827	0.1636	0.060*	
C23	0.2986 (5)	0.3098 (3)	0.1421 (3)	0.0618 (12)	
H23	0.3590	0.2391	0.1474	0.074*	
C24	0.1689 (5)	0.3270 (3)	0.1245 (3)	0.0562 (11)	
H24	0.1415	0.2690	0.1164	0.067*	
C25	0.0787 (4)	0.4309 (4)	0.1189 (3)	0.0542 (10)	
H25	−0.0102	0.4432	0.1070	0.065*	
C26	0.1194 (4)	0.5168 (3)	0.1309 (3)	0.0463 (9)	
H26	0.0570	0.5858	0.1295	0.056*	
C31	0.3048 (3)	0.7054 (3)	0.0206 (2)	0.0328 (7)	
C32	0.2915 (4)	0.6632 (3)	−0.0513 (3)	0.0430 (9)	
H32	0.2832	0.5901	−0.0361	0.052*	
C33	0.2906 (4)	0.7293 (4)	−0.1451 (3)	0.0540 (10)	
H33	0.2794	0.7011	−0.1926	0.065*	
C34	0.3062 (4)	0.8366 (3)	−0.1691 (3)	0.0537 (10)	
H34	0.3068	0.8803	−0.2326	0.064*	
C35	0.3209 (4)	0.8786 (3)	−0.0989 (3)	0.0524 (10)	
H35	0.3321	0.9508	−0.1148	0.063*	
C36	0.3189 (4)	0.8133 (3)	−0.0038 (3)	0.0433 (9)	
H36	0.3271	0.8427	0.0437	0.052*	
C41	0.0368 (3)	0.9939 (3)	0.3172 (2)	0.0302 (7)	
C42	0.0004 (4)	1.1104 (3)	0.3034 (3)	0.0411 (8)	
H42	0.0479	1.1553	0.2573	0.049*	
C43	−0.1053 (4)	1.1592 (3)	0.3575 (3)	0.0457 (9)	
H43	−0.1293	1.2370	0.3475	0.055*	
C44	−0.1755 (4)	1.0938 (3)	0.4262 (3)	0.0491 (10)	
H44	−0.2455	1.1268	0.4636	0.059*	
C45	−0.1417 (4)	0.9795 (3)	0.4393 (3)	0.0487 (9)	
H45	−0.1905	0.9354	0.4848	0.058*	
C46	−0.0356 (3)	0.9293 (3)	0.3853 (2)	0.0387 (8)	
H46	−0.0131	0.8517	0.3951	0.046*	
C51	0.1781 (3)	1.0222 (2)	0.1304 (2)	0.0301 (7)	
C52	0.0624 (4)	1.0633 (3)	0.0812 (3)	0.0438 (9)	
H52	−0.0163	1.0452	0.1109	0.053*	
C53	0.0641 (4)	1.1315 (3)	−0.0127 (3)	0.0516 (10)	
H53	−0.0140	1.1589	−0.0454	0.062*	
C54	0.1780 (4)	1.1589 (3)	−0.0578 (3)	0.0515 (10)	
H54	0.1782	1.2041	−0.1209	0.062*	
C55	0.2930 (4)	1.1189 (3)	−0.0090 (3)	0.0473 (9)	
H55	0.3712	1.1377	−0.0392	0.057*	
C56	0.2931 (3)	1.0513 (3)	0.0842 (2)	0.0363 (8)	
H56	0.3715	1.0248	0.1165	0.044*	
C61	0.3312 (3)	0.9188 (3)	0.2940 (2)	0.0362 (8)	
C62	0.3421 (4)	0.9981 (4)	0.3328 (3)	0.0518 (10)	
H62	0.2660	1.0544	0.3421	0.062*	
C63	0.4655 (5)	0.9950 (5)	0.3584 (3)	0.0730 (14)	
H63	0.4720	1.0479	0.3860	0.088*	
C64	0.5777 (5)	0.9131 (5)	0.3424 (3)	0.0753 (16)	
H64	0.6607	0.9116	0.3585	0.090*	
C65	0.5697 (4)	0.8343 (4)	0.3034 (3)	0.0675 (14)	
H65	0.6468	0.7798	0.2923	0.081*	
C66	0.4461 (4)	0.8355 (3)	0.2802 (3)	0.0497 (10)	
H66	0.4398	0.7802	0.2553	0.060*	
N1	0.3869 (4)	0.3971 (3)	0.5087 (3)	0.0552 (10)	
N2	0.1933 (3)	0.4553 (2)	0.4313 (2)	0.0374 (7)	
O	0.2771 (3)	0.2643 (2)	0.4627 (2)	0.0635 (8)	
P1	0.30183 (8)	0.62370 (7)	0.14657 (6)	0.03201 (19)	
P2	0.17669 (8)	0.92148 (7)	0.25006 (6)	0.03023 (19)	
Br	−0.05776 (3)	0.67299 (3)	0.31620 (3)	0.04306 (11)	
Ag	0.17151 (3)	0.73006 (2)	0.258501 (19)	0.03744 (9)	
S	0.28951 (9)	0.61787 (8)	0.43976 (7)	0.0431 (2)	
H1A	0.450 (4)	0.408 (3)	0.529 (3)	0.052*	
H1B	0.382 (4)	0.331 (3)	0.517 (3)	0.052*	
H2	0.128 (4)	0.515 (3)	0.404 (3)	0.052*	

### Atomic displacement parameters (Å^2^)

**Table:**

	*U*^11^	*U*^22^	*U*^33^	*U*^12^	*U*^13^	*U*^23^
C1	0.0368 (18)	0.046 (2)	0.0252 (17)	−0.0178 (16)	−0.0045 (14)	−0.0012 (15)
C2	0.054 (2)	0.042 (2)	0.038 (2)	−0.0211 (19)	−0.0063 (17)	−0.0033 (17)
C3	0.067 (3)	0.055 (3)	0.068 (3)	−0.031 (2)	−0.023 (2)	−0.008 (2)
C11	0.0319 (17)	0.0379 (19)	0.038 (2)	−0.0045 (15)	−0.0027 (15)	−0.0193 (16)
C12	0.046 (2)	0.077 (3)	0.038 (2)	0.004 (2)	−0.0008 (18)	−0.019 (2)
C13	0.060 (3)	0.094 (4)	0.049 (3)	0.015 (3)	−0.020 (2)	−0.033 (3)
C14	0.050 (3)	0.077 (3)	0.108 (4)	0.001 (2)	−0.040 (3)	−0.051 (3)
C15	0.039 (2)	0.055 (3)	0.112 (4)	−0.015 (2)	−0.012 (3)	−0.018 (3)
C16	0.038 (2)	0.044 (2)	0.065 (3)	−0.0131 (17)	−0.0085 (19)	−0.0074 (19)
C21	0.0371 (18)	0.0353 (18)	0.0341 (19)	−0.0126 (15)	0.0032 (15)	−0.0106 (15)
C22	0.042 (2)	0.039 (2)	0.066 (3)	−0.0064 (17)	−0.0017 (19)	−0.0162 (19)
C23	0.073 (3)	0.032 (2)	0.076 (3)	−0.010 (2)	0.005 (2)	−0.020 (2)
C24	0.072 (3)	0.047 (2)	0.060 (3)	−0.035 (2)	0.017 (2)	−0.022 (2)
C25	0.051 (2)	0.064 (3)	0.061 (3)	−0.030 (2)	0.004 (2)	−0.024 (2)
C26	0.040 (2)	0.043 (2)	0.059 (2)	−0.0098 (17)	−0.0002 (18)	−0.0212 (19)
C31	0.0282 (16)	0.0327 (17)	0.0338 (18)	−0.0023 (14)	−0.0004 (14)	−0.0112 (14)
C32	0.051 (2)	0.039 (2)	0.039 (2)	−0.0110 (17)	−0.0039 (17)	−0.0128 (17)
C33	0.057 (3)	0.061 (3)	0.047 (2)	−0.010 (2)	−0.0082 (19)	−0.023 (2)
C34	0.054 (2)	0.053 (3)	0.040 (2)	−0.006 (2)	−0.0013 (19)	−0.0033 (19)
C35	0.065 (3)	0.037 (2)	0.050 (2)	−0.0157 (19)	0.000 (2)	−0.0058 (18)
C36	0.051 (2)	0.037 (2)	0.045 (2)	−0.0139 (17)	−0.0014 (17)	−0.0160 (17)
C41	0.0274 (16)	0.0327 (17)	0.0319 (17)	−0.0072 (13)	−0.0041 (13)	−0.0113 (14)
C42	0.044 (2)	0.0351 (19)	0.044 (2)	−0.0126 (16)	−0.0033 (17)	−0.0086 (16)
C43	0.050 (2)	0.035 (2)	0.053 (2)	−0.0031 (17)	−0.0079 (19)	−0.0191 (18)
C44	0.045 (2)	0.064 (3)	0.046 (2)	−0.011 (2)	0.0046 (18)	−0.032 (2)
C45	0.048 (2)	0.063 (3)	0.041 (2)	−0.026 (2)	0.0097 (18)	−0.0187 (19)
C46	0.046 (2)	0.0371 (19)	0.0350 (19)	−0.0133 (16)	0.0032 (16)	−0.0136 (15)
C51	0.0344 (17)	0.0227 (15)	0.0321 (17)	−0.0071 (13)	−0.0026 (14)	−0.0071 (13)
C52	0.038 (2)	0.049 (2)	0.044 (2)	−0.0086 (17)	−0.0066 (16)	−0.0130 (18)
C53	0.053 (2)	0.050 (2)	0.047 (2)	−0.0028 (19)	−0.022 (2)	−0.0093 (19)
C54	0.073 (3)	0.041 (2)	0.035 (2)	−0.012 (2)	−0.008 (2)	−0.0046 (17)
C55	0.053 (2)	0.042 (2)	0.042 (2)	−0.0199 (18)	0.0059 (18)	−0.0045 (17)
C56	0.0356 (18)	0.0343 (18)	0.039 (2)	−0.0118 (15)	−0.0044 (15)	−0.0078 (15)
C61	0.0359 (18)	0.0413 (19)	0.0273 (17)	−0.0137 (16)	−0.0052 (14)	−0.0003 (15)
C62	0.051 (2)	0.064 (3)	0.045 (2)	−0.022 (2)	−0.0082 (18)	−0.014 (2)
C63	0.079 (3)	0.092 (4)	0.061 (3)	−0.048 (3)	−0.023 (3)	−0.010 (3)
C64	0.056 (3)	0.094 (4)	0.067 (3)	−0.039 (3)	−0.033 (2)	0.018 (3)
C65	0.040 (2)	0.069 (3)	0.070 (3)	−0.017 (2)	−0.014 (2)	0.017 (2)
C66	0.038 (2)	0.046 (2)	0.053 (2)	−0.0088 (17)	−0.0085 (18)	0.0016 (18)
N1	0.057 (2)	0.046 (2)	0.059 (2)	−0.0193 (18)	−0.0294 (18)	0.0071 (17)
N2	0.0371 (16)	0.0326 (16)	0.0386 (17)	−0.0117 (13)	−0.0124 (13)	0.0014 (13)
O	0.069 (2)	0.0395 (16)	0.078 (2)	−0.0117 (15)	−0.0264 (17)	−0.0056 (15)
P1	0.0298 (4)	0.0328 (5)	0.0343 (5)	−0.0073 (4)	−0.0003 (4)	−0.0129 (4)
P2	0.0297 (4)	0.0281 (4)	0.0315 (5)	−0.0083 (3)	−0.0012 (3)	−0.0068 (3)
Br	0.02998 (18)	0.0510 (2)	0.0468 (2)	−0.01327 (16)	−0.00403 (15)	−0.00963 (17)
Ag	0.03723 (15)	0.03262 (15)	0.04341 (17)	−0.01155 (11)	0.00531 (11)	−0.01416 (12)
S	0.0478 (5)	0.0414 (5)	0.0426 (5)	−0.0164 (4)	−0.0160 (4)	−0.0054 (4)

### Geometric parameters (Å, º)

**Table:**

C1—N1	1.308 (4)	C36—H36	0.9300
C1—N2	1.373 (4)	C41—C46	1.377 (4)
C1—S	1.683 (4)	C41—C42	1.397 (4)
C2—O	1.216 (4)	C41—P2	1.825 (3)
C2—N2	1.377 (4)	C42—C43	1.376 (5)
C2—C3	1.492 (5)	C42—H42	0.9300
C3—H3A	0.9600	C43—C44	1.373 (5)
C3—H3B	0.9600	C43—H43	0.9300
C3—H3C	0.9600	C44—C45	1.372 (5)
C11—C16	1.369 (5)	C44—H44	0.9300
C11—C12	1.393 (5)	C45—C46	1.385 (5)
C11—P1	1.828 (3)	C45—H45	0.9300
C12—C13	1.381 (6)	C46—H46	0.9300
C12—H12	0.9300	C51—C56	1.383 (4)
C13—C14	1.360 (7)	C51—C52	1.385 (4)
C13—H13	0.9300	C51—P2	1.833 (3)
C14—C15	1.358 (7)	C52—C53	1.388 (5)
C14—H14	0.9300	C52—H52	0.9300
C15—C16	1.372 (5)	C53—C54	1.361 (6)
C15—H15	0.9300	C53—H53	0.9300
C16—H16	0.9300	C54—C55	1.375 (5)
C21—C26	1.385 (5)	C54—H54	0.9300
C21—C22	1.388 (5)	C55—C56	1.378 (5)
C21—P1	1.822 (3)	C55—H55	0.9300
C22—C23	1.383 (5)	C56—H56	0.9300
C22—H22	0.9300	C61—C62	1.371 (5)
C23—C24	1.359 (6)	C61—C66	1.390 (5)
C23—H23	0.9300	C61—P2	1.823 (3)
C24—C25	1.377 (6)	C62—C63	1.388 (6)
C24—H24	0.9300	C62—H62	0.9300
C25—C26	1.379 (5)	C63—C64	1.372 (7)
C25—H25	0.9300	C63—H63	0.9300
C26—H26	0.9300	C64—C65	1.357 (7)
C31—C36	1.377 (5)	C64—H64	0.9300
C31—C32	1.390 (5)	C65—C66	1.385 (5)
C31—P1	1.827 (3)	C65—H65	0.9300
C32—C33	1.377 (5)	C66—H66	0.9300
C32—H32	0.9300	N1—H1A	0.84 (4)
C33—C34	1.377 (6)	N1—H1B	0.84 (4)
C33—H33	0.9300	N2—H2	0.89 (4)
C34—C35	1.369 (6)	P1—Ag	2.4807 (9)
C34—H34	0.9300	P2—Ag	2.4657 (9)
C35—C36	1.392 (5)	Br—Ag	2.6588 (5)
C35—H35	0.9300	Ag—S	2.8789 (10)
			
N1—C1—N2	117.2 (3)	C44—C43—H43	119.8
N1—C1—S	123.2 (3)	C42—C43—H43	119.8
N2—C1—S	119.6 (3)	C45—C44—C43	119.6 (3)
O—C2—N2	122.8 (3)	C45—C44—H44	120.2
O—C2—C3	122.6 (4)	C43—C44—H44	120.2
N2—C2—C3	114.5 (3)	C44—C45—C46	120.5 (4)
C2—C3—H3A	109.5	C44—C45—H45	119.7
C2—C3—H3B	109.5	C46—C45—H45	119.7
H3A—C3—H3B	109.5	C41—C46—C45	120.3 (3)
C2—C3—H3C	109.5	C41—C46—H46	119.8
H3A—C3—H3C	109.5	C45—C46—H46	119.8
H3B—C3—H3C	109.5	C56—C51—C52	118.6 (3)
C16—C11—C12	118.6 (3)	C56—C51—P2	122.6 (2)
C16—C11—P1	123.5 (3)	C52—C51—P2	118.6 (3)
C12—C11—P1	117.5 (3)	C51—C52—C53	119.9 (3)
C13—C12—C11	120.0 (4)	C51—C52—H52	120.1
C13—C12—H12	120.0	C53—C52—H52	120.1
C11—C12—H12	120.0	C54—C53—C52	121.1 (4)
C14—C13—C12	120.1 (4)	C54—C53—H53	119.4
C14—C13—H13	119.9	C52—C53—H53	119.4
C12—C13—H13	119.9	C53—C54—C55	119.3 (4)
C15—C14—C13	119.9 (4)	C53—C54—H54	120.4
C15—C14—H14	120.0	C55—C54—H54	120.4
C13—C14—H14	120.0	C54—C55—C56	120.4 (3)
C14—C15—C16	120.8 (5)	C54—C55—H55	119.8
C14—C15—H15	119.6	C56—C55—H55	119.8
C16—C15—H15	119.6	C55—C56—C51	120.8 (3)
C11—C16—C15	120.4 (4)	C55—C56—H56	119.6
C11—C16—H16	119.8	C51—C56—H56	119.6
C15—C16—H16	119.8	C62—C61—C66	118.9 (3)
C26—C21—C22	118.5 (3)	C62—C61—P2	124.0 (3)
C26—C21—P1	117.7 (3)	C66—C61—P2	116.9 (3)
C22—C21—P1	123.6 (3)	C61—C62—C63	120.7 (4)
C23—C22—C21	119.9 (4)	C61—C62—H62	119.7
C23—C22—H22	120.0	C63—C62—H62	119.7
C21—C22—H22	120.0	C64—C63—C62	119.3 (5)
C24—C23—C22	121.2 (4)	C64—C63—H63	120.4
C24—C23—H23	119.4	C62—C63—H63	120.4
C22—C23—H23	119.4	C65—C64—C63	121.2 (4)
C23—C24—C25	119.4 (4)	C65—C64—H64	119.4
C23—C24—H24	120.3	C63—C64—H64	119.4
C25—C24—H24	120.3	C64—C65—C66	119.6 (5)
C24—C25—C26	120.3 (4)	C64—C65—H65	120.2
C24—C25—H25	119.9	C66—C65—H65	120.2
C26—C25—H25	119.9	C65—C66—C61	120.4 (4)
C25—C26—C21	120.6 (4)	C65—C66—H66	119.8
C25—C26—H26	119.7	C61—C66—H66	119.8
C21—C26—H26	119.7	C1—N1—H1A	121 (3)
C36—C31—C32	118.9 (3)	C1—N1—H1B	118 (3)
C36—C31—P1	118.8 (3)	H1A—N1—H1B	121 (4)
C32—C31—P1	122.3 (3)	C1—N2—C2	127.6 (3)
C33—C32—C31	120.2 (3)	C1—N2—H2	114 (2)
C33—C32—H32	119.9	C2—N2—H2	118 (2)
C31—C32—H32	119.9	C21—P1—C31	102.81 (15)
C34—C33—C32	120.7 (4)	C21—P1—C11	106.31 (15)
C34—C33—H33	119.7	C31—P1—C11	104.27 (15)
C32—C33—H33	119.7	C21—P1—Ag	117.41 (11)
C35—C34—C33	119.7 (4)	C31—P1—Ag	115.68 (10)
C35—C34—H34	120.2	C11—P1—Ag	109.20 (11)
C33—C34—H34	120.2	C61—P2—C41	107.17 (15)
C34—C35—C36	120.0 (4)	C61—P2—C51	101.85 (14)
C34—C35—H35	120.0	C41—P2—C51	104.63 (14)
C36—C35—H35	120.0	C61—P2—Ag	111.47 (12)
C31—C36—C35	120.6 (3)	C41—P2—Ag	114.18 (10)
C31—C36—H36	119.7	C51—P2—Ag	116.42 (10)
C35—C36—H36	119.7	P2—Ag—P1	124.52 (3)
C46—C41—C42	118.7 (3)	P2—Ag—Br	118.59 (2)
C46—C41—P2	117.8 (2)	P1—Ag—Br	108.96 (2)
C42—C41—P2	123.5 (3)	P2—Ag—S	96.12 (3)
C43—C42—C41	120.4 (3)	P1—Ag—S	106.75 (3)
C43—C42—H42	119.8	Br—Ag—S	95.21 (2)
C41—C42—H42	119.8	C1—S—Ag	104.87 (12)
C44—C43—C42	120.4 (3)		
			
C16—C11—C12—C13	3.0 (6)	C26—C21—P1—Ag	53.9 (3)
P1—C11—C12—C13	−169.7 (3)	C22—C21—P1—Ag	−129.8 (3)
C11—C12—C13—C14	−1.0 (7)	C36—C31—P1—C21	170.3 (3)
C12—C13—C14—C15	−1.9 (7)	C32—C31—P1—C21	−8.7 (3)
C13—C14—C15—C16	2.8 (7)	C36—C31—P1—C11	−78.9 (3)
C12—C11—C16—C15	−2.1 (6)	C32—C31—P1—C11	102.0 (3)
P1—C11—C16—C15	170.1 (3)	C36—C31—P1—Ag	41.1 (3)
C14—C15—C16—C11	−0.7 (7)	C32—C31—P1—Ag	−138.0 (3)
C26—C21—C22—C23	2.0 (6)	C16—C11—P1—C21	120.5 (3)
P1—C21—C22—C23	−174.4 (3)	C12—C11—P1—C21	−67.2 (3)
C21—C22—C23—C24	0.5 (7)	C16—C11—P1—C31	12.2 (3)
C22—C23—C24—C25	−1.5 (7)	C12—C11—P1—C31	−175.4 (3)
C23—C24—C25—C26	0.0 (6)	C16—C11—P1—Ag	−112.0 (3)
C24—C25—C26—C21	2.5 (6)	C12—C11—P1—Ag	60.4 (3)
C22—C21—C26—C25	−3.5 (6)	C62—C61—P2—C41	−27.7 (3)
P1—C21—C26—C25	173.1 (3)	C66—C61—P2—C41	157.2 (3)
C36—C31—C32—C33	−0.9 (5)	C62—C61—P2—C51	81.9 (3)
P1—C31—C32—C33	178.2 (3)	C66—C61—P2—C51	−93.2 (3)
C31—C32—C33—C34	1.6 (6)	C62—C61—P2—Ag	−153.3 (3)
C32—C33—C34—C35	−0.9 (6)	C66—C61—P2—Ag	31.6 (3)
C33—C34—C35—C36	−0.5 (6)	C46—C41—P2—C61	−106.6 (3)
C32—C31—C36—C35	−0.5 (5)	C42—C41—P2—C61	73.0 (3)
P1—C31—C36—C35	−179.6 (3)	C46—C41—P2—C51	145.7 (3)
C34—C35—C36—C31	1.2 (6)	C42—C41—P2—C51	−34.6 (3)
C46—C41—C42—C43	0.5 (5)	C46—C41—P2—Ag	17.3 (3)
P2—C41—C42—C43	−179.2 (3)	C42—C41—P2—Ag	−163.1 (2)
C41—C42—C43—C44	0.5 (6)	C56—C51—P2—C61	18.1 (3)
C42—C43—C44—C45	−1.5 (6)	C52—C51—P2—C61	−167.6 (3)
C43—C44—C45—C46	1.5 (6)	C56—C51—P2—C41	129.6 (3)
C42—C41—C46—C45	−0.5 (5)	C52—C51—P2—C41	−56.1 (3)
P2—C41—C46—C45	179.2 (3)	C56—C51—P2—Ag	−103.3 (3)
C44—C45—C46—C41	−0.5 (6)	C52—C51—P2—Ag	70.9 (3)
C56—C51—C52—C53	0.3 (5)	C61—P2—Ag—P1	−75.65 (12)
P2—C51—C52—C53	−174.1 (3)	C41—P2—Ag—P1	162.73 (11)
C51—C52—C53—C54	0.1 (6)	C51—P2—Ag—P1	40.57 (12)
C52—C53—C54—C55	−0.5 (6)	C61—P2—Ag—Br	138.88 (12)
C53—C54—C55—C56	0.4 (6)	C41—P2—Ag—Br	17.26 (12)
C54—C55—C56—C51	0.0 (5)	C51—P2—Ag—Br	−104.90 (11)
C52—C51—C56—C55	−0.4 (5)	C61—P2—Ag—S	39.53 (12)
P2—C51—C56—C55	173.8 (3)	C41—P2—Ag—S	−82.09 (11)
C66—C61—C62—C63	−0.3 (6)	C51—P2—Ag—S	155.75 (12)
P2—C61—C62—C63	−175.3 (3)	C21—P1—Ag—P2	−164.49 (13)
C61—C62—C63—C64	1.5 (7)	C31—P1—Ag—P2	−42.71 (13)
C62—C63—C64—C65	−1.0 (7)	C11—P1—Ag—P2	74.48 (13)
C63—C64—C65—C66	−0.7 (7)	C21—P1—Ag—Br	−16.24 (13)
C64—C65—C66—C61	2.0 (6)	C31—P1—Ag—Br	105.53 (12)
C62—C61—C66—C65	−1.5 (5)	C11—P1—Ag—Br	−137.27 (12)
P2—C61—C66—C65	173.9 (3)	C21—P1—Ag—S	85.51 (13)
N1—C1—N2—C2	−6.3 (5)	C31—P1—Ag—S	−152.72 (12)
S—C1—N2—C2	171.7 (3)	C11—P1—Ag—S	−35.52 (12)
O—C2—N2—C1	−3.1 (6)	N1—C1—S—Ag	146.3 (3)
C3—C2—N2—C1	177.6 (4)	N2—C1—S—Ag	−31.6 (3)
C26—C21—P1—C31	−74.3 (3)	P2—Ag—S—C1	178.26 (12)
C22—C21—P1—C31	102.0 (3)	P1—Ag—S—C1	−52.88 (13)
C26—C21—P1—C11	176.4 (3)	Br—Ag—S—C1	58.72 (12)
C22—C21—P1—C11	−7.2 (4)		

### Hydrogen-bond geometry (Å, º)

**Table:**

*D*—H···*A*	*D*—H	H···*A*	*D*···*A*	*D*—H···*A*
N1—H1*A*···S^i^	0.84 (4)	2.74 (4)	3.524 (4)	158 (3)
N1—H1*B*···O	0.84 (4)	1.99 (4)	2.642 (5)	135 (4)
N2—H2···Br	0.89 (4)	2.52 (4)	3.402 (3)	174 (3)

Symmetry code: (i) −*x*+1, −*y*+1, −*z*+1.
